# Synergistic pathways to psychosis: understanding developmental risk and resilience factors

**DOI:** 10.1038/s41386-025-02261-6

**Published:** 2025-10-10

**Authors:** Kathleen J. O’Brien, Zeeshan M. Huque, Madeline R. Pike, Emma C. Smith, Nicole L. Davies, Lauren M. Ellman

**Affiliations:** Temple University, Department of Psychology and Neuroscience, Philadelphia, PA USA

**Keywords:** Human behaviour, Immunochemistry, Risk factors

## Abstract

Psychotic disorders are complex and debilitating conditions that arise from the interplay of genetic and environmental factors. A wealth of research has identified various factors that confer risk for psychosis, while comparatively less work has focused on identifying protective or resilience-promoting factors that contribute to positive outcomes in the context of psychosis risk. Given the significant heterogeneity of outcomes for individuals at risk for psychosis, it is clear that both risk and resilience factors should be considered. In this narrative review, we synthesize current research on early adversity occurring across pre-/perinatal periods, childhood, and early adolescence, which confer risk for psychosis. We also discuss several protective factors and interventions that may buffer against the effects of early adversity, thus mitigating risk and promoting improved outcomes. By integrating findings across these domains, we underscore the importance of a developmental and multidimensional approach to understanding pathways to psychosis, which may inform future directions for prevention and intervention efforts.

## Introduction

Psychotic disorders, such as schizophrenia, are debilitating psychiatric conditions characterized by the presence of positive symptoms (e.g., delusions, hallucinations), negative symptoms (e.g., reductions in motivation, pleasure, and expressiveness), and/or disorganized symptoms (e.g., unusual or disorganized thoughts, speech, or motor behavior), and often include a range of cognitive impairments (e.g., reductions in attention, memory, and mental processing) [1, 2]. Despite advances in treatment, living with a psychotic disorder is associated with reduced life expectancy [3–5], greater incidence of homelessness and poverty [6–8], and poorer overall quality of life [9]. Therefore, significant efforts have been made towards identifying factors that increase risk for developing psychosis, with the hope that early intervention may prevent, postpone, or improve illness onset [10].

Although psychotic disorders typically emerge in late adolescence or early adulthood, subtle signs and symptoms often appear much earlier in development. Increasing evidence suggests that psychotic disorders have neurodevelopmental origins, beginning as early as the prenatal period, which are influenced by a dynamic interplay between genetic vulnerabilities and environmental exposures [11–13]. Thus, adopting a developmental framework is critical for understanding how and when adverse exposures exert their greatest influence. For example, the majority of human brain neurons are generated during the prenatal period [14], with the complexity of cortical neurons increasing rapidly during the first few years of life [15]. Adolescence represents another period of heightened neuroplasticity, in which the onset of puberty acts as a catalyst for key neurobiological changes, such as synaptic pruning and myelination [16, 17], as well as reorganization of neural circuits involved in executive functioning, reward processing, and social cognition [18–21]. These early years represent vulnerable periods in which neurodevelopment is heavily influenced by environmental inputs and individual experiences [22]. A prominent conceptual model that guides this literature in the context of psychosis is the diathesis-stress model [13, 23], which posits that individuals possess varying levels of biological vulnerability that interact with environmental stressors to determine the likelihood of developing a psychiatric disorder. Together, these frameworks highlight the importance of examining environmental influences during vulnerable periods of development to understand the trajectory of psychosis risk. Thus, in this review, we focus specifically on two categories of early life adversity: prenatal and obstetric complications and environmental exposures during childhood and adolescence. These forms of adversity were selected because they occur during vulnerable windows of brain development, are consistently linked to altered neurobiological pathways implicated in psychosis, and often precede the emergence of early signs of risk [12, 24–27].

While this review emphasizes prenatal and early life exposures, we do not discount the importance of genetic contributions to psychosis risk, which are well established [28]. Rather, we aim to highlight how early environmental factors contribute to neurodevelopmental vulnerability, both independently and as modifiers of genetic risk. Moreover, increasing evidence suggests that prenatal exposures themselves can shape neurobiological predispositions, influencing postnatal brain development and stress–response systems [29]. Nonetheless, where relevant, we highlight how these prenatal and early life exposures interact with underlying genetic vulnerabilities to provide a more integrated understanding of psychosis risk.

Critically, not all individuals who are exposed to risk factors during prenatal and early life periods go on to develop psychosis later in life. Even among individuals identified as at clinical high risk (CHR) for psychosis (i.e., individuals who experience attenuated psychotic symptoms, brief intermittent psychotic episodes, and/or a genetic risk for psychosis combined with functional decline; [30]), rates of transition to a threshold psychotic disorder range between 20 and 30% [31, 32], with some individuals experiencing full remission of psychotic symptoms [33–35]. A resilience-based framework, which complements traditional risk-focused models, emphasizes how some individuals resist illness, achieve recovery, or experience positive outcomes despite adversity [36]. Within this framework, resilience is a superordinate process across development that mitigates the impact of risk and improves prognosis. It arises through the interaction of *protective factors* (which are typically risk-dependent and protect against negative outcomes) and *promotive factors* (which more broadly encompass factors that enhance psychological well-being) across biological, psychological, and social domains [36]. Related terms include *resilience factors* and *modifiable risk factors*, as even established risks may be altered through intervention. Critically, these are not simply the absence of risk factors, but distinct influences that may modify psychosis risk independently or interactively. While a growing body of literature has begun to identify protective and promotive factors in the context of psychosis risk [26, 37–39], much remains to be discussed regarding the potential dynamic interactions between risk and protective factors during early life.

Thus, in this narrative review, we explore the complex interplay between various forms of early life adversity and potential protective factors in the development of psychosis. We focus on outcomes for both individuals with psychotic disorders and those identified as CHR, which represent a well-characterized population for the prospective study of risk and protective processes [40, 41]. Specifically, we discuss how various forms of early adversity and resilience-promoting factors contribute to a range of psychosis-related outcomes, including symptom severity, functional impairment, and transition to psychosis. We also explore the underlying mechanisms of these relationships, such as stress neurobiology and structural and functional brain changes. It is important to note that many of the risk and protective factors included in this chapter are transdiagnostic and are not exclusively related to psychosis, but are associated with vulnerability to other neurodevelopmental and psychiatric disorders (e.g., depression, anxiety, attention-deficit/hyperactivity disorder, and autism spectrum disorders), which are frequently comorbid with psychosis [42, 43]. Here, we review the literature linking various risk and protective factors to psychosis-related outcomes and, where indicated, provide evidence for specificity to psychosis. However, it is important to keep in mind that these factors may reflect broader disruptions in neurodevelopmental processes that can lead to a range of clinical outcomes. Finally, by adopting a developmental framework and examining the influences of early adversity and protective factors at vulnerable periods of development, from prenatal exposures through childhood and adolescence, we aim to provide an integrative overview of pathways implicated in psychosis, which highlights converging evidence and identifies further avenues for early intervention and prevention efforts.

## Search strategy

To guide the scope of this narrative review, we focused on early life adversities and protective factors most consistently linked to psychosis risk in empirical research, particularly those supported by both human and preclinical evidence. These include pre- and perinatal exposures (e.g., maternal stress, inflammation, and obstetric complications) and early environmental stressors (e.g., trauma, peer victimization, neighborhood disadvantage, and substance use). This selection reflects a developmentally informed approach aimed at highlighting modifiable pathways to inform early intervention efforts. A search of peer-reviewed articles in English was conducted on PubMed and PsycINFO that examined each factor when experienced during early life, which we define as encompassing pre-/perinatal periods, childhood, and adolescence, in relation to risk for a psychotic disorder (e.g., schizophrenia, schizoaffective disorder, schizophreniform disorder). We included observational and intervention studies in CHR individuals to encompass research on both the modification of risk for transition to a psychotic disorder and the promotion of positive outcomes. Additionally, intervention studies conducted in individuals with psychotic disorders were also included as they related to improved outcomes (e.g., decreased symptom severity, improved functioning). Studies that examined subclinical psychotic-like experiences (PLEs) were excluded to limit the scope of the review. This is because PLEs are more common in the general population, particularly during childhood and adolescence, and because they are associated with increased risk for a range of psychopathology beyond psychotic disorders [44, 45]. In contrast, the CHR state represents an operationalized diagnostic construct with established utility for identifying risk of transition to psychosis.

## Developmental risk factors for psychosis: influences of early adversity

### Pre- and perinatal development: obstetric complications

As supported by Barker’s “Developmental Origins of Health and Disease” (DOHaD) [46], the pre- and perinatal periods are critical windows for fetal brain development, during which perturbations can have lasting effects on offspring psychopathology, also known as “fetal programming” [47–50]. Barker proposed that adverse conditions during critical periods of fetal development “program” long-term physiological changes that affect disease risk later in life. Since then, research has consistently shown that obstetric complications (OCs), broadly defined here as deviations from the typical course of pregnancy, labor, and delivery, and early neonatal development [51], are associated with an increased risk of psychotic disorders in offspring [24, 49, 52]. Importantly, OCs have also been associated with increased risk for a range of other cognitive, emotional, and behavioral difficulties, including anxiety [53], depression [54–56], ADHD [57], and autism spectrum disorder [58], indicating that their effects are not limited to pathways leading to psychosis. Although a series of OCs have been associated with schizophrenia [52], this section focuses on maternal stress, inflammation and infections, hypoxia-associated OCs and other associated pre-and perinatal factors, given that they occur at a high frequency in the population, have been found to influence each other, and have the most studies linking them to psychosis outcomes [24, 25, 49].

### Prenatal maternal stress

Prenatal maternal stress (PNMS) is a well-documented risk factor for offspring psychopathology and disease [59–61], including psychotic disorders [25, 49, 62]. Ecological studies have consistently linked events presumed to be stressful during pregnancy to offspring schizophrenia, including famine [63, 64], warfare and genocide [65–69], natural disasters [70, 71], and loss of a spouse or close relative [72, 73]. However, ecologic studies suffer from the assumption that maternal stress occurred based on events that are believed to be stressful for an entire population, but often can co-occur with other potential teratogens (e.g., malnutrition) and/or did not induce stress [74]. Investigations on individual-level stressors in human pregnancy and offspring psychosis have ranged from stressful life events [75, 76], natural disasters [77, 78], the loss of a spouse [72, 73], and poverty [79], to measures of maternal daily life stress [62] and “unwanted” pregnancy [80]. A recent review of 33 studies on PNMS and offspring schizophrenia risk found predominantly positive associations in 26 studies [81].

Because psychosocial stress engages multiple bodily systems [82], PNMS may increase offspring psychosis risk through various pathways. The hypothalamic-pituitary-adrenal (HPA; i.e., the body’s primary stress–response system) axis is the most commonly proposed mechanism in non-pregnant samples [82], though it functions differently in pregnant populations. During pregnancy, a positive feedback loop between the maternal adrenal cortex and placenta increases cortisol and placental corticotropin-releasing hormone (CRH; derived entirely from the placenta in maternal blood sera during pregnancy). However, early in pregnancy, only a portion of cortisol reaches the fetus because the placental enzyme 11β-hydroxysteroid dehydrogenase-2 (11β-HSD-2) converts much of this cortisol to inactive cortisone, and its activity declines as pregnancy progresses [83, 84]. These hormonal changes support fetal organ development, parturition, and labor activation [85–87]. Pregnant women may also become increasingly *desensitized* to the effects of stress across pregnancy, reporting less stress in response to the same event during late pregnancy compared to those who experienced it in the first trimester [88]. Nevertheless, evidence suggests that both elevations in maternal cortisol and reported maternal stress are associated with adverse birth outcomes associated with schizophrenia [25, 62, 83, 89] as well as other psychopathologies, including depression [90], and neurodevelopmental conditions such as ADHD [91] and ASD [92].

Other hypothesized indirect pathways linking PNMS to offspring psychosis outcomes include interactions with OCs, such as fetal hypoxia (reviewed in later sections) [25]. Maternal and fetal genetic vulnerabilities have also been proposed as contributing factors, as certain genetic predispositions may interact with PNMS to amplify risk for psychosis [93]. PNMS has also been linked to premorbid cognitive changes, such as lower childhood IQ [94], executive functioning [95], and language abilities [96], as well as impairments in motor skills [97]; all of which have been identified prior to psychosis onset [49]. Similarly, PNMS has been linked to reduced gray matter and hippocampal and DA abnormalities seen in psychosis [98, 99]. Collectively, these findings suggest that PNMS contributes to neurodevelopmental outcomes, beginning at birth, that increase the likelihood of psychosis, as well as cognitive and neural alterations found in the course of psychosis.

### Prenatal maternal infection and inflammation

Prenatal maternal infections and inflammation have become an important target of investigation for their role in offspring development of psychotic disorders [100]. Population-based ecological studies first provided evidence of these associations, finding increased risk of psychosis in offspring of those born during influenza epidemics compared to other periods [101–104]. Birth cohort studies further examined this association using prenatal medical records and/or analyses of prenatal blood sera that determined infections through antibodies [11, 100, 105, 106]. Based on these studies, findings indicated that multiple maternal infections during pregnancy were associated with an increased risk of offspring psychotic disorders, including respiratory infections, influenza, herpes simplex virus type 2, toxoplasma gondii, genital/reproductive infections, rubella, and bacterial infections [107–113], which have been extensively reviewed elsewhere [105, 114].

Many infections do not appear to cross the placenta [115]; therefore, one proposed mechanism linking maternal infections during pregnancy to offspring psychosis is maternal immune responses to infection, particularly elevations in proinflammatory proteins [115]. Increases in proinflammatory proteins during gestation (e.g., cytokines and chemokines), herein referred to as prenatal maternal inflammation (PNMI), have been repeatedly implicated in offspring development of psychosis [109, 116–119]. Specifically, interleukins (IL)-8, IL-6, IL-1β, TNF-α, and C-reactive protein (CRP) at various times of gestation have all been linked to an increased risk of psychotic disorders in offspring [109, 116, 118–120]. PNMI has been linked to increased inflammatory markers in the placenta and/or fetal circulation [121, 122], and there is also evidence that some maternal cytokines can cross the placenta [115, 121, 123].

The association between PNMI and offspring psychotic disorders may be explained, in part, by altered fetal neurodevelopment [117]. Preclinical research has found prenatal maternal immune activation (MIA) to be related to several neurological phenotypes found in schizophrenia (e.g., increased sensitivity to dopamine (DA) receptor agonists, decreased levels of serotonin, reductions in hippocampal and amygdala cortical volume, microgliosis, and increased microglial density in areas such as the hippocampus) [124, 125]. Similarly, rodent models of MIA have found cognitive deficits in offspring mirroring symptoms commonly seen in psychotic disorders (e.g., problems with executive functioning, cognitive flexibility, and sensory motor gating) [125]. In humans, PNMI has been associated with decreased left entorhinal cortex volumes, increased ventricular cerebrospinal fluid, and poorer performance on assessments of verbal memory and executive function, specifically in offspring with schizophrenia [115, 126–128]. Among offspring who go on to develop psychosis, there is evidence that cases with a history of prenatal infections have a more severe premorbid period, characterized by lower birth weight and poorer performance on cognitive tests in childhood, as well as a more severe course of the disorder, characterized by poorer cognitive performance and fine-motor coordination, and increased cavum septum pellucidum (CSP) length, a well-documented brain anomaly in psychosis [126, 127, 129]. Finally, PNMI may indirectly increase offspring psychosis risk through its interaction with other prenatal insults, such as PNMS and hypoxia-associated OCs, discussed elsewhere in this review [100, 117, 130, 131].

### Hypoxia-associated obstetric complications

Hypoxia-associated OCs (herein referred to as fetal hypoxia) are defined by complications that involve oxygen deprivation to the fetus and serve as one of the primary OCs that have been linked to offspring risk for developing a psychotic disorder, with evidence suggesting that as many as 20–30% of cases of schizophrenia have a history of fetal hypoxia [38, 130]. Fetal hypoxia can occur at various stages of pregnancy and labor/delivery [132] and can include prenatal complications such as preeclampsia and/or complications occurring during birth (e.g., umbilical cord problems obstructing blood flow) [133–135]. Fetal hypoxia is associated with an earlier age of onset and a worsened premorbid period, characterized by childhood motor and cognitive difficulties [52, 136–140]. Importantly, many of these findings also differentiated unaffected siblings from cases who developed schizophrenia, suggesting that the interaction between fetal hypoxia and liability for schizophrenia portended a worsened course of the disorder [12, 138, 140]. Findings suggest that fetuses who later go on to develop schizophrenia, compared to control fetuses, exhibit a reduced neurotrophic response to hypoxia (assessed through brain-derived neurotrophic factor levels in cord blood) [141]. Although these results did not directly test genetic mechanisms, other studies have highlighted how genes implicated in schizophrenia (e.g., AKT1, BDNF) may render the fetus more vulnerable to hypoxic insult [142, 143]. For instance, genome-wide derived polygenic risk scores for schizophrenia were related to a higher prevalence of OCs [144]. The genes related to these OCs were also overexpressed in placentas from complicated pregnancies compared to control pregnancies, particularly when the fetuses were male [144]. Thus, genetic risk for psychosis may render the fetus more susceptible to OCs, increasing the probability of adverse neurodevelopmental outcomes.

There is also evidence that other OCs (like infection and PNMS) can increase the likelihood of fetal hypoxia. For instance, a review of the relationship between infection, proinflammatory cytokines, and hypoxia in the fetal brain indicated that infection exposure and proinflammatory cytokines may alter the threshold at which hypoxia becomes neurotoxic, increasing vulnerability to lesser hypoxic events [145]. Likewise, PNMS has been linked to increased rates of asphyxia during birth and decreased Apgar scores (rating newborn health after birth), both of which are used to assess the construct of hypoxia-associated OCs [146]. Taken together, evidence suggests that fetal hypoxia is a prominent OC linked to psychosis that has been found to interact with both risk genes and other OCs associated with psychotic disorders Nevertheless, challenges remain in understanding the hypoxia to psychosis pathway due to heterogeneous definitions of fetal hypoxia across existing studies, as well as methodological challenges in measuring hypoxia during pregnancy [12, 38, 89].

### Maternal health behaviors during pregnancy

A range of prenatal substance exposures (e.g., nicotine, alcohol, and cannabis) and health behaviors (e.g., nutrition, sleep) have been explored in their link to offspring psychosis. Evidence has linked maternal smoking during pregnancy, measured via early-gestation serum cotinine levels, to increased schizophrenia risk, as well as greater severity of negative symptoms and reality distortion (e.g., hallucinations) in offspring who develop the disorder [147, 148]. Additionally, rodent models have linked Δ⁹-tetrahydrocannabinol (THC) exposure to epigenetic changes that mediate synaptic development [149]. This may offer insight into the mechanisms underlying human cohort findings linking prenatal cannabis use to increased risk of psychosis in offspring [150]. While evidence directly connecting maternal alcohol use to offspring schizophrenia is limited, high levels of maternal alcohol use during pregnancy are associated with OCs, such as low birth weight and umbilical cord vasoconstriction that elevate schizophrenia risk [151]. Animal studies further support that in utero exposures to nicotine, alcohol, and cannabis are associated with neural and behavioral outcomes found in schizophrenia [152].

Additionally, PNMS warrants consideration in relation to health behaviors during pregnancy due to evidence linking heightened PNMS with engagement in smoking, decreased exercise, unhealthy eating, and further adverse lifestyle behaviors [11, 153]. Adverse health behaviors are also associated with OCs (e.g., vitamin D deficiency and preeclampsia) that have been identified in association with offspring psychosis risk [11, 154]. The bidirectional nature of adverse health behaviors and PNMS suggests that either may serve as mediators in models of fetal programming following prenatal risk factors [155]. Recent reviews have highlighted associations between elevated maternal pre-pregnancy body mass index (BMI), obesity, and/or nutritional deficits (e.g., iron, vitamin D) with offspring psychosis-related outcomes [25, 38]. Nutritional intake’s relationship to fetal outcomes may also be understood through its role in inflammatory status and placental dysfunction, as nutrient deficiencies have been linked to immune system and CNS functioning in offspring [25, 156]. Similarly, maternal sleep disturbances have been linked to elevated levels of inflammatory markers, such as CRP and IL-6 [157], and may represent a potential mediator in the relationship between PNMS and fetal programming [155]. Consequently, modifiable risk factors, such as maternal health behaviors, may contribute to the observed associations between OCs and psychosis, potentially influencing fetal programming through interactions with inflammation, nutrition, sleep, and other lifestyle factors.

### The role of offspring sex and timing of prenatal exposure

Research suggests that the effects of pre- and perinatal insults on offspring development likely vary based on both fetal sex and gestational timing. For example, male fetuses are thought to be at higher risk for mortality and morbidity in response to PNMS exposure, also known as the viability–vulnerability tradeoff hypothesis [87]. In line with this, exposure to prenatal maternal daily life stress has been shown to have a two-fold increase in the odds of psychotic disorders, primarily in male offspring [62]. Similarly, PNMI contributions to offspring outcomes are likely also dependent on fetal sex [54, 118, 158, 159]. Some studies report a greater risk for psychotic disorders in male offspring after PNMI or maternal infection, which may contribute to the male-biased prevalence of psychotic disorders [118, 160]. Similarly, animal studies have indicated greater reductions in learning task performance in male offspring following fetal hypoxia [161], which is bolstered by findings that schizophrenia risk genes in placentae may be associated with upregulation when the fetus is male. However, other findings suggest that chronic fetal hypoxia may be associated with cognitive difficulties (e.g., decreased verbal IQ, inhibition) in females [144, 162], suggesting that future research is warranted to better assess sex differences in offspring outcomes following fetal hypoxia.

Timing of prenatal exposures also may differentially contribute to psychosis risk. While PNMS exposure during the second trimester has been most consistently associated with offspring risk for psychosis, evidence is limited, and it still remains inconclusive regarding which trimester presents the highest vulnerability [25]. Offspring risk for psychotic disorders has been linked to infection or PNMI exposure at various times in gestation, suggesting inconsistencies in a gestational vulnerable period for psychosis risk [74, 106, 109, 118, 163, 164].

In summary, pre- and perinatal factors interact with each other, as well as with susceptibility genes [144, 165], to play a critical role in shaping offspring vulnerability to psychosis. These effects may be moderated by both the timing of exposure and fetal sex, though findings remain mixed. Importantly, not all individuals exposed to OCs go on to develop psychosis; rather, it has been proposed that such early insults may function as a “primer,” increasing the brain’s sensitivity to subsequent postnatal stressors [166]. In the following section, we examine early life environmental stressors that may build upon pre-existing vulnerabilities and further contribute to the development of psychosis.

### Early life: environmental risk factors

Experiences during childhood and adolescence can exert profound, long-lasting effects on mental health trajectories, including risk for psychosis. A growing body of research highlights how adverse early life exposures interact with neurodevelopmental processes to shape brain circuits involved in systems associated with psychosis, including stress regulation, reward processing, and social cognition [167]. Here, we focus on specific forms of environmental exposures during childhood and adolescence that have been consistently associated with heightened psychosis risk: childhood trauma, peer victimization, and neighborhood disadvantage [24, 168–171]. In addition, adolescent substance use represents a particularly potent risk factor for psychosis during a time of heightened neuroplasticity, changes to reward circuitry, and increased susceptibility to social influences [18, 19, 172–174]. In this section, we synthesize evidence on these key psychosocial and environmental risk factors, emphasizing both epidemiological associations and emerging neurobiological mechanisms.

### Childhood trauma

Childhood trauma, defined as experiences of abuse and or neglect which result in actual or potential harm to a child’s health, development, and/or dignity (WHO, 2024), has consistently been associated with increased risk for developing psychosis [169, 175, 176]. Meta-analytic findings suggest 80–90% of individuals at CHR have been exposed to at least one type of childhood trauma [170, 177]. Repeated evidence suggests that childhood trauma is associated with greater symptom severity for individuals at CHR [177–179] and those with psychotic disorders [175, 176, 180–183]. Moreover, among individuals with psychosis, histories of childhood trauma are associated with a range of negative outcomes, including poorer cognitive functioning [184, 185], poorer social and role functioning [186–189], poorer treatment response [190, 191], and lower symptom remission [192, 193].

Notably, recent models of childhood trauma have highlighted distinct dimensions of deprivation and threat, which differentially impact neural, cognitive, and emotional development [194–197]. As such, increasing attention has been focused on parsing the specific effects of different types of childhood trauma in the context of psychosis [198–202], though findings have been mixed. Although there is some evidence that threat-based traumas, such as physical and sexual abuse, may be associated with the largest increased odds of developing a psychotic disorder [200, 201], several studies have concluded that all types of childhood trauma are associated with increased risk, with exposure to multiple traumas increasing risk in a linear relationship [198, 201, 202]. Further, the specific effects of childhood trauma types may be difficult to differentiate as multiple types of trauma commonly co-occur [203, 204]. Moreover, recent evidence suggests that childhood neglect modulates neural profiles of abuse, such that individuals with high levels of both abuse and neglect show a qualitatively different pattern of neural activation, compared to those with high levels of either type of trauma alone [205].

### Peer victimization

Peer victimization (e.g., bullying) has repeatedly been associated with a greater risk for developing a psychotic disorder [168, 206–208]. Critically, the relationship between peer victimization and psychotic symptoms remains after accounting for a range of other factors, including family adversity, comorbid psychopathology, and other negative life events [209]. A prospective cohort study provided evidence that victimization is associated with the development of a psychotic disorder in a dose-response fashion, with victims of sibling bullying being 2–3 times more likely to meet criteria for a psychotic disorder, and victims of both sibling and peer bullying having more than four times the odds of developing a psychotic disorder [168]. According to the social defeat hypothesis, repeated social exclusion has deleterious effects on DA functioning, heightening sensitivity of the mesolimbic DA pathway and increasing vulnerability to psychosis [210, 211]. It has also been suggested that certain pre-existing difficulties, such as cognitive impairments and non-specific effects of socioeconomic disadvantage, precede experiences of bullying and contribute to heightened risk of victimization [212], increasing risk for psychosis in a bidirectional and compounding manner [213]

### Neighborhood disadvantage

Beyond individual-level adverse exposures, neighborhood-level factors may also contribute to risk for psychosis. Neighborhood disadvantage, which refers to growing up in impoverished conditions with limited access to social and economic resources, has been studied extensively in the context of psychosis, though findings are mixed. Some studies have suggested that individuals exposed to lower socioeconomic conditions at birth or during childhood and adolescence are at increased risk for the onset of a psychotic disorder in adulthood [214–218], while others found no or opposite associations [219]. However, the literature examining neighborhood disadvantage is complex, as this risk factor often encompasses other neighborhood-level factors, such as urbanicity, which has also been related to increased risk for psychotic disorders [214, 220, 221]. Other neighborhood conditions associated with increased risk for psychotic disorders include increased exposure to environmental toxins and lack of green space [24, 221, 222], and social disorganization, which itself encompasses a range of additional neighborhood conditions, including crime levels, single-person households, residential mobility, ethnic density, and voter turnout [223]. While it may be difficult to disentangle the precise risk conferred by each of these interrelated neighborhood-level factors, chronic exposure to multiple disadvantaged neighborhood conditions may be associated with greater cumulative stress [24, 224], likely converging on several neurobiological systems, discussed in later sections.

### Adolescent substance use

As highlighted previously, adolescence is a vulnerable neurodevelopmental period characterized by heightened neuroplasticity and brain maturation [172–174], as well as identity formation, increased autonomy, and sensitivity to social influences [18, 225]. Thus, adolescents are more likely to engage in risk-taking behaviors, such as substance use [226], which has been identified as a primary predictor of transition to psychosis [227, 228]. During adolescence, the brain is particularly vulnerable to the effects of substance use, with cannabis frequently studied in relation to psychosis risk [228–231]. Moreover, individuals with a history of early life adversity are also at greater risk for initiation of substance use [232]. Earlier initiation and more chronic substance use during adolescence are also associated with a greater magnitude of risk for psychotic disorders [231, 233]. Some studies suggest that psychosis-spectrum symptoms may precede the initiation of substance use, with substances used to cope with distressing symptoms [234] or early life stressors [235]. However, evidence from prospective, longitudinal studies has found that the associations between adolescent substance use and risk for psychosis onset exist even after controlling for psychosis symptoms that preceded substance use [236, 237].

### Biopsychosocial mechanisms: cascade of risk

As highlighted previously, the risk factors discussed here do not occur in isolation. While these exposures vary in content and timing, they likely converge through shared and overlapping mechanisms that unfold across development. Beginning in the prenatal period, exposure to stressors and environmental insults may initiate a cascade of interconnected neurobiological disruptions, cognitive changes, and behaviors that could set the stage for increased vulnerability to subsequent adversity during childhood and adolescence [11].

PNMI and other OCs may act as a neurodevelopmental ‘primer’, leading to subtle brain, immune, and hormonal alterations that can influence subsequent neurodevelopment [115, 238, 239]. OCs have been linked to a variety of cognitive difficulties [49, 240], which may in turn influence social functioning (e.g., interactions with peers, teachers, family members), potentially resulting in additional difficulties [55]. For example, evidence suggests that cognitive difficulties and other developmental risk factors increase the risk of being bullied and victimized by peers, which in turn increases the risk for developing psychosis [212, 213]. Additionally, stressors during prenatal and childhood periods have been found to prime the HPA-axis for exaggerated reactivity, increasing sensitivity to future stressful events, and increasing the likelihood that daily events will be perceived as stressful [241, 242]. In particular, elevated stress sensitivity may play a role in the early stages of psychosis development, as greater emotional reactivity to minor stressors has been associated with increases in subtle psychotic experiences [242]. Consistent with this, individuals at CHR tend to be more reactive to stressful life events, compared to controls [243, 244]. Moreover, compared to non-converters, CHR individuals who transitioned to psychosis exhibit a blunted cortisol awakening response, further indicating HPA-axis dysregulation [245]. Additionally, HPA-axis dysregulation is linked to disruptions in sleep patterns [246–248], which is another established risk factor for psychosis [249]. Sleep disturbances, in turn, further exacerbate cognitive dysfunction, increase inflammation, and are associated with increases in stress reactivity [157, 250, 251], creating a self-perpetuating cycle that contributes to vulnerability.

Over time, repeated stress exposure may initiate further neurobiological disruptions, such as altered DA signaling. For example, evidence suggests that dysregulated stress responses, likely in combination with genetic predispositions [252, 253], directly influence the DA system, contributing to hyperactivity of the mesolimbic DA pathway [254, 255]. DA dysregulation may be further compounded by substance use during adolescence, in part, through indirect effects of the endocannabinoid system on DA signaling [256]. DA dysregulation is a hallmark of psychosis [257–259], such that higher levels of DA in the striatum have been repeatedly observed and may amplify the salience of irrelevant stimuli, contributing to the development of delusions and hallucinations—a concept often referred to as “aberrant salience” [260]. Critically, these processes may unfold in a cascading and self-reinforcing manner. For example, early experiences of cognitive difficulties and peer victimization may further exacerbate stress sensitivity and increase risk for initiating substance use.

Additionally, it has been hypothesized that chronic HPA-axis activation may impair the regulation of glucocorticoids (GCs), leading to persistent elevations in GC levels [261–263]. Both chronic GC exposure and DA dysregulation may interact with and contribute to other biological processes, such as increased production of proinflammatory cytokines [264, 265] and excessive glutamatergic signaling, which has been associated with glutamate excitotoxicity [266, 267]. Together, these interactive processes have been proposed to contribute to progressive structural and functional brain alterations by disrupting normal developmental processes and contributing to decreased neurogenesis, impaired synaptic plasticity, and cell death [268–270]. Notably, many of the relationships described here are based on correlational findings or theoretical models. While these patterns offer insight into possible developmental mechanisms, additional longitudinal and mechanistic research is needed to delineate causality and interaction effects.

In line with this cascading model, it should be noted that early life adversity, including prenatal insults, may not exert immediate effects but instead alter the developmental trajectory of brain maturation in ways that only become apparent during later critical periods. For example, during adolescence, a time of significant neural and psychosocial changes [16–18, 225], previously latent vulnerabilities may be unmasked, leading to the emergence of psychosis symptoms [271, 272]. Specifically, evidence of reduced synaptic connectivity in schizophrenia derives from postmortem studies [273–276], and consistent findings of cortical gray matter reductions in individuals with psychosis [277–279] and CHR [280–282]. Accelerated gray matter loss during adolescence has also been linked to increased psychosis risk, suggesting that normative developmental processes may interact with prior insults to drive neurobiological change [271, 283]. Although genetic factors have been linked to increased pruning in schizophrenia [284], it has been proposed that early adversity could reduce baseline synaptic density or compromise neural organization, thereby lowering the threshold for pruning-related disruptions to precipitate psychotic symptoms [285]. Support for this model comes from preclinical studies indicating that a fetal hippocampal lesion is associated with schizophrenia-like brain pathology that emerges with increased pruning [286, 287], as well as other neurodevelopmental processes [288], which occur during adolescence. However, pre- and postnatal adversities are not focal events that result in lesions, but rather represent interacting exposures that likely impact multiple neurodevelopmental systems over time. Thus, pruning abnormalities are unlikely to represent the sole pathway through which early exposures confer risk for psychosis. For more comprehensive discussions of the various neurobiological processes linking prenatal and early life adversity with psychosis risk, we direct the readers to several excellent reviews [27, 115, 288–290]

Although OCs and early life stress increase risk for a range of psychopathologies [50, 54, 291], there is some evidence for specificity to psychosis. For example, our group has provided evidence that fetal hypoxia is associated with decreased birth weight among individuals with schizophrenia, but not affective psychosis (i.e., mood disorders with psychotic features) [292]. Additionally, trauma and stress in childhood are linked to subthreshold psychosis symptoms beyond their shared correlates, including comorbid psychopathology [293–295]. Further, common psychological sequelae of early adversity, such as anxiety and depression, may mediate this relationship, representing an affective pathway to psychosis [242, 296, 297]. Nevertheless, it is plausible that many of these early experiences increase risk for shared phenotypes across disorders (e.g., brain alterations, cognitive difficulties), but future studies are necessary to determine factors that contribute to the specificity of results. Importantly, these risk factors also offer opportunities for early identification and studies examining resilience in the face of early adversity, discussed further in our sections on protective factors.

## Protective and promotive influences in psychosis: mitigating the effects of developmental risk factors

A resilience-based approach helps conceptualize how some individuals experience variable outcomes despite exposure to risk factors. This not only relates to rates of transition to psychosis, but also improved prognosis if psychosis occurs - manifesting in lower symptom severity, better functioning, and greater capacity for recovery [36, 298]. Central to this framework are protective and promotive factors, as well as protective and preventative interventions, which are not dependent on risk presence and typically introduced before possible risk exposure. Importantly, protective factors are not necessarily the absence or inverse of risk factors; though some, such as reduced maternal stress during pregnancy, are supported by intervention studies. Consequently, several studies have tested the efficacy of interventions based on these protective factors, which we review separately along with preventative interventions.

### Pre-/perinatal protective and promotive factors

#### Serious infection reduction and responses to infection

Given that most pregnant individuals experience some type of infection during the course of their pregnancies, it is important to understand the differential impact of various infections during pregnancy [299]. The severity of infection, rather than exposure alone, might explain some of the variability in the impact of prenatal infections on offspring [74, 83]. However, there is evidence for individual genetic variation in maternal responses to infection [12, 106, 115]. For example, immune-related genetic polymorphisms in the IL-1 complex or TNF-alpha are associated with greater basal levels of proinflammatory cytokines and an increased inflammatory response to infection [12, 115]. It is possible that individuals with these and other genetic profiles are more vulnerable to the effects of prenatal infection, therefore increasing offspring risk for psychosis. Conversely, individuals with genetic profiles that promote more effective regulation of immune responses may be buffered against the adverse effects of infection, highlighting that stronger or more adaptive immune functioning could serve as a protective factor even prior to intervention. However, the relationship between specific genetic polymorphisms and prenatal immune response in relation to offspring risk for psychotic disorders has yet to be examined [12, 106, 115].

#### Maternal health behaviors during pregnancy

Several prenatal maternal health behaviors have received attention as possible protective factors with theoretical links to modified risk for psychosis. For example, prenatal nutritional deprivation has been linked to an increased risk of offspring psychotic disorders, suggesting enriched maternal nutrition may have the potential to reduce risk. Sufficient intake of folic acid and iron prenatally, crucial for preventing maternal anemia and fetal hypoxia, may be protective [300, 301]. In addition, prenatal plasma-free choline and a choline-rich diet during pregnancy have been linked to increased offspring cognitive functioning in infancy and childhood [302–304]. Higher fetal choline concentrations are also related to increases in alpha-7 nicotinic receptor activation, receptors that are typically highly expressed in the fetus and critical for development [303]. Further, genetic deficiencies in alpha-7 nicotinic receptors have been linked to the development of psychotic disorders [303]. Some evidence suggests that greater omega-3 fatty acid docasahexaenoic acid (DHA) levels in the offspring at birth are associated with better birth outcomes and improvements in cognitive, visual, and motor development in infancy [305, 306].

Additionally, some studies suggest that psychological factors like an optimistic disposition during pregnancy reduce the risk of preterm birth, which has independently been linked to changes in offspring development and an increased psychosis risk [307, 308]. Maternal optimism is also associated with the use of positive coping strategies during pregnancy [309, 310], with strategies like positive appraisal during pregnancy found to be related to lower levels of emotional distress [311]. These are relevant protective factors given the relationship between PNMS and psychosis risk discussed previously in this review.

#### Pre-/perinatal interventions

Intervention research conducted during pregnancy and early life has included both protective and preventative efforts. Based on research identifying maternal infection severity during pregnancy and offspring psychosis risk [312], offspring vaccination has been robustly tested and recommended as a preventative intervention. The Centers for Disease Control and Prevention (CDC) recommends that pregnant women receive the influenza vaccine, with research from large birth cohorts finding no adverse effects in offspring [312, 313], and studies have observed fewer instances of influenza illness in pregnant individuals who are vaccinated [312–314]. Although prenatal vaccination is also effective for promoting maternal and fetal health, longitudinal studies are needed to examine its potential in protecting against offspring development of psychiatric illnesses associated with prenatal infections, including psychotic disorders. Other preventative interventions that have been studied include dietary supplements during pregnancy. One randomized control trial demonstrated that infants born to a high prenatal choline diet had an inhibited P50 response (i.e., intact sensory gating) at 5 weeks old compared to placebo-treated infants [302–304], with P50 deficits consistently found in schizophrenia and, to an extent, in individuals at CHR [315]. Given mounting evidence in support of other nutrition and dietary supplements like those described above, studying maternal nutrition interventions during pregnancy as a preventative against psychosis represents a fruitful direction for further research. Additionally, poor sleep quality during pregnancy has been linked to differences in child development in areas of global cognition, brain structure, and behavioral problems, with sleep interventions (e.g., Cognitive Behavioral Therapy for Insomnia; CBT-I, mindfulness) found to improve sleep difficulties during pregnancy [316–318]. Although the efficacy of preventative prenatal sleep interventions has not yet been examined in the context of offspring development, it is another promising avenue of exploration for promoting resilience against developmental factors that may give rise to later psychopathology.

Numerous protective interventions during pregnancy have focused on targeting maternal mental health, specifically reducing maternal stress. Several randomized control trials (RCTs) have tested stress management interventions, cognitive behavioral therapy (CBT), and positive psychological interventions that are effective in decreasing maternal perceived stress and depression symptoms during pregnancy, with some studies also finding decreases in diurnal salivary cortisol [319–322]. Longitudinal follow-up studies with offspring are critical to explore any potential long-term benefits of protecting against the development of psychosis. Additionally, there is evidence to suggest that postnatal factors may promote offspring resilience after exposure to maternal prenatal stress, including parental sensitivity and environmental enrichment [323]. RCTs examining parental sensitivity and skin-to-skin contact interventions (e.g., Kangaroo Care) find widespread benefits to the infant and child (e.g., increased newborn brain structural connectivity, decreased cortisol and autonomic nervous system reactivity, and improved cognitive and language development), emphasizing the potential of resilience-promoting postnatal environments to protect against the effects of PNMS [323]. Lastly, increasing attention has been paid to understanding the importance of modifying exposures to adverse neighborhood and environmental factors during pregnancy. Research has linked exposure to adverse environmental conditions to elevations in inflammation during pregnancy, including higher levels of air pollution and other potential toxicants (e.g., plastics) [305, 324]. Therefore, potential protective interventions could include reducing exposure to ambient air pollution (e.g., not exercising outdoors during periods of high pollution, if living in urban areas), breathing through the nose when outside, having a high quality air purifier, and reducing exposure to plastics (e.g., frequently vacuuming and not drinking out of plastic bottles) (U.S. Environmental Protection Agency, 2019).

### Childhood and adolescent protective and promotive factors

#### Psychological and emotional factors

Broader psychological processes—such as coping strategies, emotion regulation, and personality traits—may mitigate the effects of adverse experiences. Coping strategies have long been implicated in the development, maintenance, and outcomes of psychotic disorders [325–329]. An individual’s ability to adapt to change or cope with stress has been associated with decreased symptom severity in CHR individuals [330, 331], as well as lower rates of transition to psychosis [332, 333]. Studies find that more frequent use of certain coping strategies (e.g., avoidance, emotion-focused) by CHR individuals is associated with greater negative symptoms and lower psychosocial functioning [334]. In comparison, more frequent use of other coping strategies like problem-solving and seeking social support has been associated with less severe negative and psychotic symptoms, though it remains unclear whether specific coping strategies impact the likelihood of transition to psychosis [334]. Nevertheless, the use of adaptive coping strategies among individuals at CHR has been associated with better social and role functioning [335], symptom severity [335, 336], and treatment response [337]. In contrast, poor emotion regulation strategies, like emotional suppression, were shown to mediate the relationship between psychosis risk and mental health symptoms like emotion dysregulation and substance use [338]. Additionally, research suggests that certain traits like optimism, an internal locus of control, and positive self-appraisal may promote resilience as they are associated with decreased perceived stress and improved functioning among individuals with psychosis [339, 340]. Some of these resilience-promoting factors may also be modifiable; for example, implementation of mindfulness and self-affirmation strategies may improve emotion regulation, reduce stress, increase positive emotions, and increase optimism [341, 342].

#### Psychosocial factors

Beyond individual-level protective factors, social and community factors may further protect against psychosis risk. Affectionate familial relationships and effective extended family networks may be a protective factor among CHR individuals [343], with caregiver affection and positive engagement during the CHR period associated with symptom reduction [344]. Family relationships high in expressed emotion (e.g., the intensity and nature of hostile or critical emotional expressions) have been linked to heightened relapse rates and poorer functioning among individuals with psychosis [345, 346], while greater levels of warmth and optimal family involvement have been associated with improved functioning and reduced symptom severity in CHR individuals [346]. Further, evidence suggests that greater perceived social support is associated with reduced risk for psychosis and improved functioning [343, 347, 348], though social network research remains limited by a lack of standardization in conceptualizing social networks across studies [349, 350]. For individuals with marginalized social identities, other community-level factors such as social inclusion, education, job and food security, and access to healthcare may be particularly protective [24]. Increased ethnic group affiliation [351] and access to community individuals with similar ethnoracial backgrounds may potentially be protective against the effects of exposure to chronic social stressors like discrimination among children and adolescents [352, 353]. On a broader scale, systemic efforts—including changes to assessment and diagnosis training, multilevel interventions, and shifts in funding priorities—may be necessary to modify psychosis risk, particularly for marginalized racial/ethnic individuals [24, 354].

### Childhood and adolescent interventions

Intervention studies conducted among children and adolescents have largely targeted lifestyle and psychosocial factors that may be protective or resilience-promoting in the context of psychosis risk. Modifying sleep habits has been one of the most studied and promising lifestyle targets. Sleep disturbances (i.e., sleep disorders, deviations in objective measures of sleep, subjective reports of poor sleep) are highly prevalent in psychosis [355–357], often precede psychosis onset, and have been found to be predictive of transition to psychosis [356, 357]; therefore, an intervention window prior to psychosis onset is emphasized. One study found CBT-I resulted in better sleep, improvements in negative affect, and reduced psychotic experiences among CHR individuals [358]. Additionally, stress and early adversity can disrupt sleep, and sleep disruptions, in turn, have been associated with cortisol reactivity and higher levels of inflammation [359–361]. Therefore, sleep improvements may buffer the effects of early adversity on mental health outcomes and represent a promising resilience-promoting factor [360].

Exercise and diet interventions have also been robustly tested as protective against psychosis. Physical activity has been linked to reduced symptom severity, improved mood, memory, psychosocial functioning, and quality of life across CHR and psychosis populations [362–365]. Further, longitudinal evidence suggests that greater physical activity levels are associated with a lower risk of transition to psychosis in those at CHR [366]. Importantly for those with a history of early adversity, exercise has been shown to buffer the effects of stress on mental health outcomes [367], likely through the modulation of multiple biological systems, such as lower overall cortisol levels [368, 369], reduced inflammation [370, 371], and increases in hippocampal neurogenesis [372, 373]. Additionally, exercise may be most beneficial in combination with a healthy diet and nutrition patterns that may reduce metabolic risk factors common among individuals with psychosis [374–376]. These combination exercise and diet interventions may further mitigate the effects of adverse exposures by reducing inflammation and regulating stress-sensitive hormonal systems [377, 378]. That said, many of the sleep, exercise, and diet studies just described were tested in late adolescence/early adult samples, suggesting a critical need for further studies that introduce these interventions earlier in development, which may help increase their efficacy in buffering against early life adversities and promoting positive outcomes.

Lastly, psychosocial interventions may help support individual-level resilience processes. An RCT revealed that a family psychoeducation approach, including training in communication and problem-solving skills, exhibits potential in improving attenuated positive symptoms in CHR individuals [379]. Similarly, another RCT suggests that reductions in perceived maternal criticism predict decreases in subthreshold positive symptoms [380]. Other clinical interventions have focused on social activation techniques, such as group sessions and constructive group activities, which have successfully decreased negative symptoms in individuals with recent-onset psychosis [381].

### Mechanisms of resilience

Interactions between behavioral protective factors and various neurodevelopmental mechanisms may also support effective adaptation and recovery from stressors across early life. For example, neural plasticity, the brain’s ability to adaptively change synaptic connections over time [382], supports the strengthening of adaptive circuits and maintenance of various cognitive functions [383–385]. Therefore, it is possible that plasticity in response to stress is protective against poor mental health outcomes by promoting compensatory neural mechanisms and buffering the effects of environmental insults [386–388]. Critically, there is significant overlap in the neural circuits most affected by stress and those implicated in risk for psychosis [389]. For example, while hippocampal volume loss is characteristic of psychosis [390–393], longitudinal evidence suggests that CHR individuals who remitted did not exhibit reductions in hippocampal CA1 volume over time [394]. Similarly, among adults who have experienced childhood maltreatment, resilient individuals (i.e., those who did not develop psychopathology) exhibited greater hippocampal volume compared to adults with psychopathology [395]. Additionally, resilient individuals showed lower hippocampal activation to emotional faces and increased amygdala habituation to stress, suggesting a greater ability to regulate emotions, dampen threat processing, and modulate stress responses [395]. In line with this, CHR individuals who remitted showed greater accuracy, greater amygdala activation, and stronger negative amygdala-prefrontal functional connectivity during an emotional faces task [396]. This suggests that adaptive or compensatory mechanisms within these circuits—such as increased synaptic efficiency, strengthened top-down regulatory control, and more effective stress–response modulation—may protect against the development of psychosis, and could inform future neurobiological intervention research in psychosis.

## Interplay between risk and resilience

**Fig. 1:**
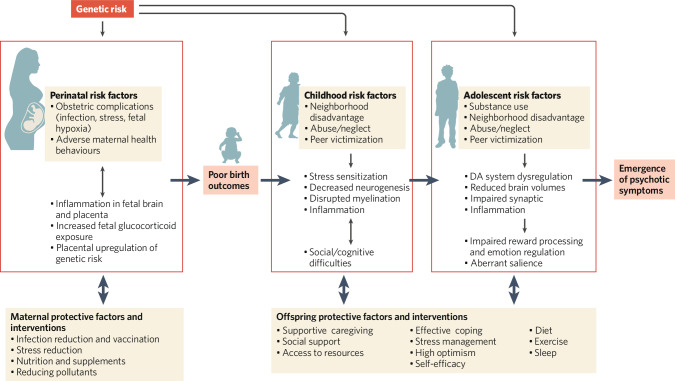
Interacting and cascading effects of biopsychosocial exposures across early development. This model highlights the various mechanisms through which genetic risk interacts with a range of prenatal and environmental stressors to contribute to or compound risk for psychosis. Additionally, several promising protective and promotive factors, as well as preventive interventions, have been identified that may buffer or counteract the effects of exposures across developmental stages.

While several of the factors reviewed here have been studied in isolation, both risk and resilience factors should be conceptualized as dynamic and interacting factors across developmental periods and biopsychosocial levels [298]. Critically, individuals vary widely in their susceptibility to environmental inputs, including both positive and negative contexts [397–400]. Therefore, those with heightened susceptibility may be particularly vulnerable to negative contexts, but also more responsive to protective factors, potentially buffering against negative outcomes. As discussed, early life adversity, such as prenatal insults and/or childhood trauma, can disrupt neurodevelopment and increase risk for psychosis by altering stress–response systems, neural circuitry, and other biological systems (e.g., immune systems). However, resilience factors may buffer against these risks by promoting adaptive coping, support, and self-efficacy. For example, evidence suggests that the presence of supportive caregivers [401, 402] and other social support [403] buffers against the risk of psychopathology following trauma exposure in childhood, including for individuals with psychosis [404]. Risk and resilience can also interact across environmental and biological levels, such that social engagement may buffer against the deleterious effects of neighborhood poverty on hippocampal volume among CHR individuals [405]. Further, the use of effective coping mechanisms can reduce distress associated with psychotic symptoms [406], which likely has downstream effects on stress sensitivity and overall stress–response [407–410]. Expanding previously existing models of psychosis risk to incorporate resilience provides a more comprehensive understanding of the complex and dynamic processes that shape outcomes for individuals at risk for psychosis. Figure 1 provides an overview of the interacting and/or compounding effects of various risk factors, while highlighting promising protective factors and interventions that have the potential to buffer against risk. These interactions have critical implications for early intervention and prevention efforts, discussed below.

## Clinical implications

Existing early intervention programs, like Coordinated Specialty Care (CSC), have demonstrated efficacy in the treatment of individuals experiencing a first-episode psychosis [411–413] through a combination of individual psychotherapy, medication management, family support, and psychoeducation (SAMHSA, 2023), and are being extended to individuals at CHR [414–416]. Meta-analytic findings also suggest that broader, non-psychosis-specific treatments, such as CBT [417] and antidepressant medications [418], may be effective in reducing transition rates and improving functional outcomes for CHR individuals, with the incorporation of psychoeducation about CHR status important to mitigate stigma-related stress [419–422]. However, these treatment models have not yet fully incorporated preventative and protective interventions into their approach. For example, CBT-I approaches could be incorporated for individuals with sleep difficulties [358], and Trauma-focused CBT (TF-CBT) strategies may also aid in decreasing both psychotic and post-traumatic stress symptoms for those with a history of trauma [423]. Mindfulness-based stress reduction (MBSR) techniques [424] could be more regularly offered to expecting mothers to mitigate exposure to maternal stress [425], as well as in children and adolescents to promote stress management. Additionally, families could engage in Family-Focused Therapy (FFT-CHR), which may improve psychotic symptoms among children and adolescents growing up in families with high emotional expression [380]. Beyond the individual-level, family-focused and culturally-informed treatment (CIT-S) has also been shown to significantly decrease psychotic symptoms [426, 427]. Effective integration of these approaches and other resilience-promoting factors discussed in this review requires individualized and targeted treatment plans for implementation at different stages of development. Additionally, understanding the systems and broader sociocultural conditions within which an individual grows up is necessary. Recent evidence finds significant socioeconomic disparities in treatment access and effectiveness of CSC programs [428], suggesting that addressing structural barriers, while challenging, will be critical to ensure early intervention efforts reach those most vulnerable. Finally, a prevention approach that aims to provide information at the population level irrespective of risk status holds the promise of improving large-scale access to education about psychosis risk and protective factors, and may be beneficial for implementation in maternal healthcare, school, and community settings [40, 429–431].

## Conclusion and future directions

Expanding upon traditional risk-based models of psychosis to incorporate resilience-promoting factors will improve our ability to identify those who will go on to develop psychosis, with the aim of preventing or postponing illness onset and improving outcomes. Examining protective factors across biopsychosocial levels, particularly in early development, may help inform the development of novel interventions and prevention efforts. Given the nascent literature on protective factors in psychosis, further empirical testing of these factors through intervention studies, as well as identification of other resilience-promoting factors, should be a priority. Still, continued research on risk factors remains essential, particularly given the heterogeneous and developmentally dynamic pathways through which psychosis emerges. For example, a growing body of literature suggests that experiencing ethnoracial discrimination is a stressful and potentially traumatic event [432, 433] that may contribute to psychosis risk both independently and in interaction with other early adversities and neighborhood conditions shaped by structural racism [24, 354, 434]. These experiences may play a role in the well-documented racial disparities observed in schizophrenia-spectrum diagnoses [435, 436]. However, there remains a notable gap in research examining ethnoracial discrimination as a developmental risk factor for psychotic disorders, particularly when experienced in childhood or adolescence. Existing studies have more often focused on its relationship with subthreshold psychotic experiences rather than diagnosable psychotic disorders during these vulnerable periods [405, 437, 438]. This review also underscores the need for future research to differentiate more clearly between risk and resilience pathways for affective versus non-affective psychoses, as many existing studies do not distinguish or compare these diagnostic categories of psychosis in relation to specific risk and resilience factors. Adopting a developmental framework to extend current findings across both risk and protective domains, as well as addressing questions of diagnostic specificity, will be essential to advancing a more precise and equitable understanding of psychosis trajectories beginning in early life.

To move towards a resilience-based approach to psychosis, several recommendations are made for future directions. Importantly, protective factors should not be viewed merely as the absence or inverse of risk, but as distinct constructs that warrant dedicated investigation [36, 298]. Research should consider person-centered approaches, including computational or predictive modeling approaches, which characterize the interactions of an individual’s unique risk and resilience factors [224]. Secondly, given the complexity of protective and risk factors interacting across multiple biopsychosocial levels, research efforts should prioritize multi-disciplinary collaboration, including involving individuals with lived experience who may offer invaluable insights [439]. Prospective longitudinal studies tracking risk-resilience interactions over time are also needed to clarify their role across critical developmental periods. Ultimately, moving towards a resilience-based framework holds promise for reshaping how we understand, prevent, and intervene in psychosis. By integrating protective factors into developmental models, we can develop more nuanced, equitable, and effective approaches to supporting individuals at risk.
